# Clinical features and outcomes of patients with stable or unstable chest pain and no-obstructive coronary artery disease

**DOI:** 10.3389/fcvm.2022.951183

**Published:** 2022-08-23

**Authors:** Nello Cambise, Alessandro Telesca, Saverio Tremamunno, Tamara Felici, Antonio De Vita, Monica Filice, Gessica Ingrasciotta, Eleonora Ruscio, Filippo Crea, Gaetano A. Lanza

**Affiliations:** Department of Cardiovascular Disease, Fondazione Policlinico Universitario A. Gemelli IRCCS, Università Cattolica del Sacro Cuore, Rome, Italy

**Keywords:** stable angina, acute coronary syndrome, coronary microvascular dysfunction, clinical outcome, non-obstructed coronary arteries

## Abstract

**Background:**

Coronary microvascular dysfunction can be responsible for both stable angina and acute coronary syndrome (ACS). There are scarce data, however, about comparisons of clinical characteristics and outcomes of these 2 groups of patients.

**Materials and methods:**

We studied 47 consecutive patients who underwent coronary angiography for angina syndromes and showed no obstructive stenosis. Patients were divided in 2 groups, according to their clinical presentation, i.e., stable angina (*n* = 21) or non-ST segment elevation ACS (NSTE-ACS; *n* = 26). An intracoronary acetylcholine (Ach) test was performed in 12 and 17 patients of the 2 groups, respectively. Angina status, assessed by Seattle Angina Questionnaire (SAQ), and clinical events were assessed after 1, 6, and 30 months. An exercise stress test was performed 1 month after discharge.

**Results:**

Clinical characteristics and exercise test results of the 2 groups were largely similar. Ach testing induced epicardial or microvascular spasm in 6 (50.0%) and 10 (58.8%) stable and NSTE-ACS patients, respectively (*p* = 0.72). Stable patients reported higher rates of angina, compared to NSTE-ACS patients, both at 1 (*p* = 0.04) and 30 months (81 vs. 50%, *p* = 0.036) of follow-up. SAQ scores were also lower in stable vs. NSTE-ACS patients. Ach testing results showed no association with clinical outcomes.

**Conclusion:**

Clinical characteristics and exercise and Ach testing results are similar in angina patients with no-obstructive coronary artery disease with a stable or NSTE-ACS presentation. Stable patients show a worse symptomatic outcome irrespective of Ach test results.

## Introduction

In recent years several studies have shown that more than a half of patients with stable angina chest pain and about 10–15% of those referred for an acute coronary syndrome (ACS) do not show any obstructive coronary artery disease (CAD) at angiography (1–3). The presence of abnormalities in coronary microcirculation can be demonstrated in a sizeable proportion of these patients, suggesting that coronary microvascular dysfunction (CMD) is involved in the origin of symptoms of these patients (4–10). However, controversial findings have been reported about clinical outcome of patients with stable angina definitely or likely related to CMD (11–14), whereas there are no well-established data about clinical outcome of patients with ACS related to coronary microvascular abnormalities (i.e., coronary microvascular spasm) (9, 15). Moreover, there are scarce data comparing clinical characteristics and outcome of patients with chronic/stable vs. acute/unstable ischemic syndromes related to CMD. Accordingly, the aim of this study was to compare clinical characteristics and outcomes of two groups of such patients.

## Methods

### Study population

Between January and December 2018, we prospectively enrolled consecutive patients who underwent a first coronary angiography at our Department of Cardiovascular Medicine because of typical angina symptoms but showed normal coronary arteries or non-obstructive CAD (no stenosis higher than 40% in any epicardial artery) at invasive angiography.

Patients were excluded from the study in case of: (1) any previous assessment with coronary angiography (including coronary computed tomographic angiography); (2) typical variant angina or documentation of transient ST-segment elevation (suggesting epicardial spasm) on the admission electrocardiogram (ECG) and/or during any angina attack; (3) a diagnosis of takotsubo syndrome or acute myocarditis, based on clinical, echocardiographic and magnetic resonance imaging; (4) any evidence of other significant cardiac disease (valvular heart disease, cardiomyopathy, etc.); (5) non-sinus rhythm (atrial fibrillation/flutter; pacemaker); (6) any ECG abnormalities that might have influenced the correct assessment of the results of the study investigation (e.g., left bundle branch block, significant ST-segment/T wave changes); (7) any clinically relevant systemic disease (e.g., acute or chronic inflammatory disease, liver failure, end-stage renal failure, malignancies).

Patients were divided in two groups according to their clinical presentation: (1) stable angina group, which included patients undergoing elective coronary angiography because of stable, predominantly exercise-induced angina, and (2) ACS group, which included patients admitted to the Emergency Department of our hospital because of a suspicion of no-ST segment elevation ACS (NSTE-ACS), with or without significant cardiac troponin raise, due to one or more episodes of chest pain at rest.

From each patient, we collected a detailed clinical history, including cardiovascular risk factors and medications. Hypertension was defined as the detection of blood pressure (BP) ≥ 140/90 mmHg or use of anti-hypertensive drugs. Hypercholesterolemia was defined as total cholesterol levels > 220 mg/dL or use of anti-cholesterolemic drugs. Diabetes mellitus was defined as the detection of glycated hemoglobin > 6% or use of antidiabetic drugs. Active smoking was defined as having smoked any cigarettes in the last 6 months.

As for clinical protocol, high-sensitivity serum cardiac troponin I (cTnI) was measured on admission in all patients. Furthermore, in patients in the ACS group, cTnI was subsequently measured after 6 and 24 h, and then every 24 h until discharge, unless differently indicated.

The study was approved by the Ethics Committee of our Institution. Patients were carefully informed of the scope and procedures of the study and gave informed written consent to participate in the study.

### Coronary angiography and acetylcholine test

Coronary angiography was performed 24–72 h after admission using a radial artery approach. Immediately after coronary angiography, that showed no obstructive CAD, an acetylcholine (Ach) provocative test was performed in a subgroup of patients, according to the operator’s discretion. Patients who underwent Ach test were free from any vasoactive therapy for at least 48 h and the test was conducted before any administration of vasoactive drugs.

Acetylcholine was administered into the left coronary artery with increasing doses of 20, 50, and 100 μg, over a period of 3 min each, and with a 2–3 min interval between injections. Coronary angiography was performed at the end of each dose, or immediately in case of induction of chest pain and/or ischemic ECG changes. The test was interrupted in case of positive findings or occurrence of any side effect.

An epicardial spasm was diagnosed in case of focal or diffuse narrowing of the epicardial coronary diameter ≥ 90%, associated with typical symptoms and/or ischemic ECG changes. Coronary microvascular spasm was diagnosed if typical symptoms and/or ischemic ECG changes were induced in the absence of epicardial spasm at angiography, as defined above (16). In case of a positive Ach test, intracoronary nitroglycerin was given to relieve the spasm.

### Clinical follow-up

The clinical status of patients was assessed 1, 6, and 30 months after discharge by clinical visits. The primary end point was a combination of clinical events, including death, acute myocardial infarction, stroke, re-hospitalization for chest pain, coronary revascularization and angina recurrence/persistence. In case of clinical events, detailed information were obtained from medical records.

Furthermore, angina status was assessed by Seattle Angina Questionnaire (SAQ), a 19-item self-administered questionnaire that explores, through five scales, clinically relevant aspects of coronary artery disease (17). Each SAQ item (physical limitation, angina frequency, angina stability, treatment satisfaction, disease perception) is scored on a 0–100 scale, with higher scores indicating better functional status.

### Exercise stress test

A symptom/sign-limited ECG exercise stress test (EST) was performed, after drug discontinuation, at 1-month follow-up, using a standard treadmill Bruce protocol. EST was stopped in cases of physical exhaustion, progressive angina (Borg scale > 6), ST-segment depression > 4 mm, or any relevant clinical events (e.g., dyspnea, hypotension, arrhythmias). The test was considered positive for myocardial ischemia when a horizontal or downsloping ST-segment depression was ≥ 1 mm was induced in one or more leads (except aVR).

### Statistical analysis

Data were analyzed with the SPSS 27.0 statistical software (IBM-SPS Italia, Inc., Florence, Italy). Due to the small number of patients included in the study, comparisons between groups of continuous variables were done by Mann–Whitney U-test. Discrete variables were compared by Fisher exact test. Data of continuous variables are reported as median with (interquartile range). A *p* < 0.05 was always required for statistical significance.

## Results

### Clinical and laboratory findings

Overall, 47 patients were enrolled in the study, 21 (45%) referred to coronary angiography because of stable angina and 26 (55%) because of a suspicion of NSTE-ACS. A typical raise and fall pattern of cTnI was detected in 11 of the latter group of patients (42%), who were therefore diagnosed as having acute myocardial infarction with no-obstructive CAD (MINOCA).

The main clinical characteristics of the 2 groups of patients are summarized in Table 1. The two groups did not significantly differ with regard to the main clinical features and cardiovascular risk factors, except for active smoking, which was more frequently found in stable patients (*p* = 0.02). Echocardiography showed normal left ventricle ejection fraction and normal regional ventricular contraction in both groups; the E/e’ ratio was normal in stable angina patients, whereas it was of borderline value in ACS patients, suggesting a possible initial left ventricle diastolic dysfunction; however, the E/A ratio was similar in the 2 groups.

**TABLE 1:** Main characteristics of the two groups of patients.

	Stable patients (*n* = 21)	ACS patients (*n* = 26)	*p*
Age (years)	60 (56–67)	59 (48–67)	0.59
Female (%)	13 (62%)	11 (42%)	0.24
BMI (Kg/m^2)^	25.2 (24.0–28.5)	26.3 (24.2–29.8)	0.37
eGFR (mL/min)	88 (71–99)	96 (84–101)	0.16
**CV risk factors**			
Hypertension	14 (67%)	15 (58%)	0.59
Dyslipidaemia	12 (57%)	11 (42%)	0.38
Diabetes	2 (9.5%)	3 (12%)	0.26
Active smokers	6 (29%)	2 (8%)	0.02
Ex-smokers	7 (33%)	4 (15%)	0.02
**Echocardiographic findings**			
LVEF (%)	60 (58–64)	61 (59–64)	0.59
E/A ratio	0.91 (0.84–0.96)	0.90 (0.76–1.1)	0.72
E/e’ ratio	6.7 (5.5–7.2)	8.0 (7.0–8.6)	0.005
**Pharmacological therapy at discharge**			
Aspirin	7 (33%)	17 (65%)	0.04
Clopidogrel	2 (9.5%)	2 (7.7%)	1.00
Beta-blockers	6 (29%)	13 (50%)	0.23
ACE-i/ARBs	11 (52%)	11 (42%)	0.56
Ca-channel blockers	11 (52%)	10 (39%)	0.39
Statins	12 (57%)	7 (27%)	0.04
Nitrates	0 (0%)	1 (4%)	1.00
Ranolazine	1 (5%)	1 (4%)	1.00
Diuretics	1 (5%)	1 (4%)	1.00
Anticoagulants	3 (14%)	0 (0%)	0.08
Antidiabetic drugs	1 (5%)	2 (8%)	1.00

BMI, body mass index; eGFR, estimated glomerular filtration rate; CV, cardiovascular; LVEF, left ventricle ejection fraction; ACE, angiotensin-converting enzyme; ARBs, angiotensin-II receptor blockers.

Pharmacological therapy at discharge was also largely similar in the two groups, although aspirin was more frequently prescribed to ACS patients (65 vs. 33%, *p* = 0.04) and statins were more frequently given to stable angina patients (57 vs. 27%, *p* = 0.04). Coronary angiography showed totally normal coronary arteries in most patients; non-significant stenoses (< 40%) were indeed only found in 4 (19%) and 7 (27%) of stable angina and ACS patients, respectively (*p* = 0.73).

Acetylcholine test was performed in 12 (57%) and 17 (65%) patients of the 2 groups, respectively (*p* = 0.76). No major adverse events occurred during the test. The maximum Ach dose administered during the test was 100 (50–100) and 50 (50–100) μg in stable and ACS group, respectively (*p* = 0.26). The test induced some form of coronary constriction in 6 (50.0%) and 10 (58.8%) patients of the 2 groups, respectively (*p* = 0.72). Specifically, microvascular spasm occurred in 4 (33%) of stable angina patients and 6 (35%) of ACS patients (*p* = 0.86), whereas an epicardial spasm occurred in 6 patients, 2 (16.7%) and 4 (23.5%) of stable and ACS groups, respectively. Positivity of the test was observed at the dose of 20 μg in 4 patients (25.0%), 50 μg in 7 (43.8%), and 100 μg in 5 (31.2%).

### Exercise stress test

Overall, 45 out 47 patients completed the ECG exercise stress test 1 month after discharge. Two patients, one in each group, were indeed unable to perform the test due to a significant impairment of effort tolerance. The main results of exercise stress test are summarized in Table 2. The test was positive in 15 patients (75%) of Group 1 and in 17 patients (68%) of Group 2 (*p* = 0.52). There were also no significant differences between the 2 groups in the other main EST variables.

**TABLE 2:** Main exercise stress test findings.

	Stable patients (*n* = 20)	ACS patients (*n* = 25)	*p*
EST result			0.52
Positive	9 (45%)	13 (52%)	
Negative	5 (25%)	8 (32%)	
Questionable	6 (30%)	4 (16%)	
**Basal**			
Systolic BP (mmHg)	125 (115–140)	125 (110–132)	0.75
Diastolic BP (mmHg)	75 (70–85)	80 (75–90)	0.23
Heart rate (bpm)	84 (74–91)	75 (68–85)	0.04
RPP (bpm × mmHg)	10640 (9360–11520)	9460 (7925–11002)	0.17
**Peak EST**			
Exercise duration (s)	420 (257–551)	429 (371–576)	0.51
Systolic BP (mmHg)	170 (140–200)	160 (150–170)	0.49
Diastolic BP (mmHg)	85 (80–90)	90 (80–95)	0.37
Heart rate (bpm)	138 (133–153)	144 (130–157)	0.79
RPP (bpm × mmHg)	24160 (19460–28980)	23715 (19920–26520)	0.70
STD max (mm)	1.75 (1.50–2.75)	1.25 (1.00–1.87)	0.65
Angina	2 (10%)	2 (8%)	0.89

EST, exercise stress test; BP, blood pressure; RPP, rate-pressure product; STD, ST-segment depression.

### Clinical outcomes

The main data of clinical follow-up and the results of SAQ at 1, 6, and 30 months in the 2 groups are summarized in Table 3.

**TABLE 3:** Clinical outcomes of the two groups of patients.

	1-month follow-up			6-month follow-up			30-month follow-up		
			
	Stable patients (*n* = 21)	ACS patients (*n* = 26)	*p*	Stable patients (*n* = 21)	ACS patients (*n* = 26)	*p*	Stable patients (*n* = 21)	ACS patients (*n* = 26)	*p*
**Major clinical events**									
Angina recurrence (%)	15 (71.4)	10 (38.5)	0.04	15 (71.4)	13 (50.0)	0.23	17 (81.0)	13 (50.0)	0.04
Hospital admission (%)	0 (0.0)	1 (3.8)	1.00	0 (0.0)	2 (7.7)	0.49	0 (0.0)	2 (7.7)	0.49
MINOCA (%)	0 (0.0)	1 (3.8)	1.00	0 (0.0)	1 (3.8)	1.00	0 (0.0)	1 (3.8)	1.00
**Seattle Angina Questionnaire**									
Physical limitation	93 (78–100)	100 (97–100)	0.06	100 (93–100)	100 (100–100)	0.02	100 (77–100)	100 (100–100)	0.02
Anginal stability	75 (50–100)	100 (69–100)	0.04	75 (50–100)	88 (25–100)	0.83	50 (50–88)	100 (100–100)	< 0.001
Anginal frequency	80 (70–100)	100 (80–100)	0.04	90 (80–100)	90 (88–100)	0.58	80 (70–100)	100 (100–100)	< 0.001
Treatment satisfaction	81 (59–91)	88 (81–100)	0.08	88 (81–100)	100 (88–100)	0.21	81 (72–88)	97 (81–100)	0.02
Disease perception	67 (58–83)	58 (56–83)	0.67	67 (58–83)	71 (58–83)	0.92	58 (50–75)	79 (77–92)	0.007

MINOCA, myocardial infarction with no-obstructive coronary artery disease.

Overall clinical events during a follow-up of 30 months occurred in 17 patients (81%) of the stable angina group and 13 (50%) of the ACS group (*p* = 0.036). Almost all events consisted of persistence/recurrence of angina chest pain. No death occurred, whereas 2 ACS patients (7.7%) underwent re-hospitalization for acute chest pain (1 with troponin raise). Stable angina patients showed a higher rate of angina recurrence compared to ACS patients already at 1-month follow-up (71 vs. 38%, *p* = 0.04).

**FIGURE 1:**
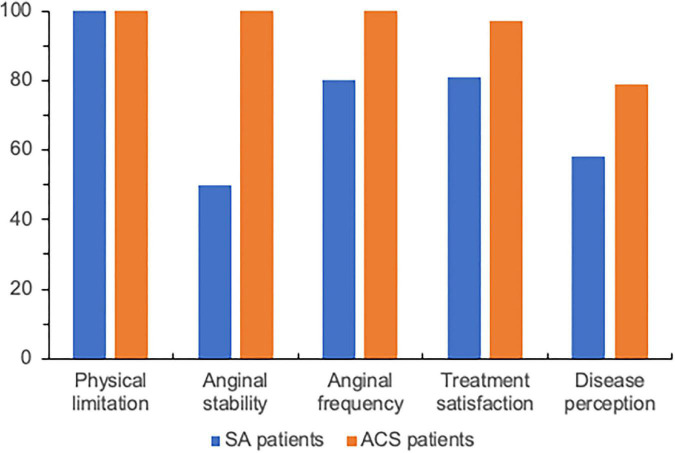
Median values of Seattle Angina Questionnaire items at 30-month follow-up in patients with stable angina (SA) or non-ST elevation acute coronary syndrome (ACS) but no obstructive coronary artery disease. See Table 3 for details of analysis.

Seattle Angina Questionnaire results were in agreement with the referred recurrence of chest pain symptoms, with stable angina patients showing lower scores, compared to ACS patients, for several items at 1-month and, most important, for all items at 30-month follow-up (Figure 1), although there were no major differences in SAQ scores between the 2 groups at the 6-month evaluation.

### Acetylcholine test results and clinical outcome

The main data of clinical follow-up and the results of SAQ at 1, 6, and 30 months in the subgroups of patients with positive or negative Ach test are summarized in Table 4.

**TABLE 4:** Clinical outcomes of patients with positive and negative Acetylcholine-test.

	1-month follow-up			6-month follow-up			30-month follow-up		
			
	Negative Ach-test (*n* = 13)	Positive Ach-test (*n* = 16)	*p*	Negative Ach-test (*n* = 13)	Positive Ach-test (*n* = 16)	*p*	Negative Ach-test (*n* = 13)	Positive Ach-test (*n* = 16)	*p*
**Major clinical events**									
Angina recurrence (%)	5 (38.5)	12 (75.0)	0.067	6 (46.2)	13 (81.3)	0.06	8 (61.5)	12 (75.0)	0.69
Hospital admission (%)	0 (0.0)	1 (6.3)	1.00	0 (0.0)	1 (6.3)	1.00	0 (0.0)	1 (6.3)	1.00
MINOCA (%)	0 (0.0)	1 (6.3)	1.00	0 (0.0)	1 (6.3)	1.00	0 (0.0)	1 (6.3)	1.00
**Seattle Angina Questionnaire**									
Physical limitation	100 (98–100)	100 (75–100)	0.12	100 (100–100)	100 (93–100)	0.06	100 (78–100)	100 (90–100)	0.82
Anginal stability	100 (50–100)	75 (50–100)	0.42	88 (44–100)	75 (25–100)	0.84	100 (69–100)	100 (50–100)	0.77
Anginal frequency	100 (80–100)	90 (70–100)	0.08	95 (75–100)	90 (80–90)	0.23	100 (86–100)	100 (80–100)	0.94
Treatment satisfaction	88 (80–89)	88 (69–100)	0.53	91 (88–100)	100 (81–100)	0.44	81 (72–89)	88 (81–100)	0.39
Disease perception	67 (56–83)	58 (17–75)	0.65	71 (58–92)	67 (42–83)	0.53	63 (50–92)	67 (50–92)	0.93

Ach, acetylcholine.

Patients with positive Ach test tended to show a higher recurrence of chest pain both at 1- and 6-month follow-up. However, there was no difference in angina recurrence at 30 months. SAQ scores did not significantly differ between patients with positive Ach-test compared with those with negative Ach-test both at 1-, 6-, and 30-month follow-up (Table 4).

## Discussion

Several interesting findings emerge from our study. First, our data show that patients with stable angina and patients admitted with a diagnosis of NSTE-ACS, but having normal or near normal epicardial coronary arteries at angiography, present largely similar clinical characteristics and results of exercise stress test and intracoronary Ach test.

However, patients presenting with stable angina showed a higher recurrence of chest pain and a greater negative impact of angina symptoms on clinical status, as assessed by SAQ, both at short- and long-term follow-up. On the other hand, the only 2 re-admissions for acute chest pain, over a follow-up period of 2.5 years, occurred in patients presenting with an ACS pattern. These results are not unexpected and might be explained by different mechanisms involved in causing chest pain symptoms in the 2 groups. While episodic, unpredictable microvascular spasm (or even epicardial spasm) might be responsible for an acute presentation, which might variably recur during follow-up in patients presenting with an ACS pattern, structural and/or functional abnormalities of coronary microcirculation, resulting in chronic impairment of microvascular dilation and/or increased constrictor activity, might instead be involved in patients with stable microvascular angina. The frequent persistence/recurrence of angina episodes, despite multiple pharmacological therapy, and the consequent negative impact on quality of life, in these patients is, indeed, a well-known finding (11–13).

Of note, our data are largely in agreement with those of Chauhan et al. who also reported a worse symptomatic outcome at a follow-up of 7–13 months in patients with the typical picture of cardiac syndrome X (stable angina, positive exercise stress test, normal coronary arteries) compared to patients presenting with a diagnosis of unstable angina and also showing normal coronary arteries (18). Interestingly, stable patients in the latter study were found to show a higher rate of increased pain perception at intracardiac manipulation of catheters and/or intracoronary injection of contrast medium, suggesting that increased cardiac pain sensitivity contributes to the worse symptomatic outcome of these patients (19–21).

A second worth noting finding of our study is that, although including a small group of patients only, no deaths or life-threatening clinical events were observed. Furthermore, only 2 recurrences of acute chest pain requiring hospitalization were observed in the ACS group over a period of 2.5 years. Thus, overall, our data confirm the benign clinical course of stable microvascular angina patients and, at the same time, suggest that severe clinical events are also rare in those presenting with NSTE-ACS.

A third relevant finding of our study is the similar results observed with Ach test in the 2 groups patients. This result seems rather challenging, as a higher rate of positivity might have been expected in the ACS group, who presented with angina chest pain at rest, suggesting either epicardial or microvascular coronary spasm. In our study, indeed, in the subgroup of patients who underwent Ach test, positivity for microvascular spasm in stable and unstable patients was 33 and 35%, respectively (*p* = 0.86), whereas an epicardial spasm was induced in only 16.7 and 23.5% of patients, respectively.

These data are partially at variance with those of some previous studies, that reported higher rates of epicardial spasm during Ach test, both in stable and ACS patients with NO-CAD. Thus, among 921 stable patients, Ong et al. found a prevalence of microvascular spasm and epicardial spasm of in 24.2 and 33.4%, respectively (7). Ohba et al. among 370 similar Asiatic patients, reported a prevalence of 13.5% (21.3% in women) and 58.4% of microvascular and epicardial spasm, respectively (22). On the other hand, Montone et al. (9), among 80 patients with suspected MINOCA, showed rates of microvascular and epicardial spasm of 16 and 30%, respectively. However, Pirozzolo et al., in 96 similar patients, found Ach test positivity for microvascular and epicardial spasm of 31 and 27%, respectively (10). The globally lower detection of epicardial spasm in our patients, compared to previous studies and ours cannot be easily explained, but it might be related to the fact that we carefully excluded patients with stigmata of variant angina in this study.

Finally, in the present study Ach test was unable to predict symptomatic outcome. In a previous study on MINOCA patients, Ach test positivity predicted a higher rate of major cardiac events and worse symptomatic outcome during long-term follow-up (7). In this study, however, most events occurred in patients with epicardial vasospasm and mainly in patients who discontinued vasodilator therapy. In the Ohba et al. study, on the other hand, no event occurred among patients with Ach positivity who continued their calcium-antagonist therapy (22). Thus, further studies seem needed to clarify the prognostic implications of NSTE-ACS caused by coronary microvascular spasm.

## Limitations of the study

Some limitations of our study should be acknowledged. First, we did not assess the endotypes of CMD in our patients (23), and the presence of CMD was assessed in a subgroup of patients only exclusively by intracoronary Ach-test. Thus, the prevalence of microvascular and epicardial spasm in response to the test might be biased by selection criteria; furthermore, we cannot draw definitive conclusions about the differences in the mechanisms of the clinical presentation between the 2 groups; however, the main scope of our article was to compare clinical characteristics and outcome of patients mainly according to clinical presentation rather than the mechanism of CMD. Second, Ach test positivity for coronary microvascular spasm was diagnosed according to reproduction of typical symptoms and/or ischemic ECG changes, in the absence of epicardial spasm at angiography; however, assessment of coronary blood flow (CBF) might have revealed a higher number of patients with CMD and possible CMD-related clinical symptoms through abnormal response to the test. Third, no intravascular imaging was performed in our patients; therefore, we cannot be sure that thromboembolic complications at the level of non-significant epicardial plaques might have been responsible for the clinical presentation in at least some ACS patients, as suggested by a recent study (24); however, there was no suspicion for some kind of plaque complication at angiographic images in any patient. Finally, the sample size of our study is rather small; thus, our data need to be confirmed in larger populations of patients.

## Conclusion

Our data show that clinical and angiographic findings, as well as responses to exercise testing and Ach testing, of patients with angina pectoris and NO-CAD presenting with a stable pattern of angina or a clinical picture of NSTE-ACS are largely similar. However, stable patients show a significant worse symptomatic outcome, compared to NSTE-ACS patients, whereas Ach test shows no impact on clinical outcome of these patients.

## Data availability statement

The raw data supporting the conclusions of this article will be made available by the authors, without undue reservation.

## Ethics statement

The studies involving human participants were reviewed and approved by the Ethics Committee Università Cattolica del Sacro Cuore. The patients/participants provided their written informed consent to participate in this study.

## Author contributions

NC, TF, and GI: patient recruitment and follow-up. AT and ST: diagnostic investigation. AD: statistical analysis. MF and ER: database handling. FC: critical review of the manuscript. GL: design of the study, statistical analysis, and manuscript preparation. All authors contributed to the article and approved the submitted version.
